# E3 ubiquitin ligase gene *BIRC3* modulates TNF-induced cell death pathways and promotes aberrant proliferation in rheumatoid arthritis fibroblast-like synoviocytes

**DOI:** 10.3389/fimmu.2024.1433898

**Published:** 2024-09-05

**Authors:** Qingliang Meng, Kai Wei, Yu Shan

**Affiliations:** ^1^ Department of Rheumatism, Henan Province Hospital of Traditional Chinese Medicine (TCM), Zhengzhou, Henan, China; ^2^ Department of Rheumatology and Immunology, Affiliated Hospital of Shandong University of Traditional Chinese Medicine, Jinan, China; ^3^ Department of Rheumatology, Shanghai Guanghua Hospital of Integrative Medicine, Shanghai University of Traditional Chinese Medicine, Shanghai, China; ^4^ Guanghua Clinical Medical College, Shanghai University of Traditional Chinese Medicine, Shanghai, China; ^5^ Institute of Arthritis Research in Integrative Medicine, Shanghai Academy of Traditional Chinese Medicine, Shanghai, China

**Keywords:** BIRC3/cIAP2, tumor necrosis factor, rheumatoid arthritis, ubiquitination, MAPK/NF-κB

## Abstract

Rheumatoid arthritis (RA) is a chronic inflammatory autoimmune disease characterized by synovitis, degradation of articular cartilage, and bone destruction. Fibroblast-like synoviocytes (FLS) play a central role in RA, producing a significant amount of inflammatory mediators such as tumor necrosis factor(TNF)-α and IL-6, which promote inflammatory responses within the joints. Moreover, FLS exhibit tumor-like behavior, including aggressive proliferation and enhanced anti-apoptotic capabilities, which collectively drive chronic inflammation and joint damage in RA. TNF is a major pro-inflammatory cytokine that mediates a series of signaling pathways through its receptor TNFR1, including NF-κB and MAPK pathways, which are crucial for inflammation and cell survival in RA. The abnormal proliferation and anti-apoptotic characteristics of FLS in RA may result from dysregulation in TNF-mediated cell death pathways such as apoptosis and necroptosis. Ubiquitination is a critical post-translational modification regulating these signaling pathways. E3 ubiquitin ligases, such as cIAP1/2, promote the ubiquitination and degradation of target proteins within the TNF receptor complex, modulating the signaling proteins. The high expression of the *BIRC3* gene and its encoded protein, cIAP2, in RA regulates various cellular processes, including apoptosis, inflammatory signaling, immune response, MAPK signaling, and cell proliferation, thereby promoting FLS survival and inflammatory responses. Inhibiting *BIRC3* expression can reduce the secretion of inflammatory cytokines by RA-FLS under both basal and inflammatory conditions and inhibit their proliferation. Although BIRC3 inhibitors show potential in RA treatment, their possible side effects must be carefully considered. Further research into the specific mechanisms of *BIRC3*, including its roles in cell signaling, apoptosis regulation, and immune evasion, is crucial for identifying new therapeutic targets and strategies.

## 1 Introduction

Rheumatoid arthritis (RA) is a chronic autoimmune condition marked by synovitis, degradation of articular cartilage, and bone destruction. A notable pathological feature is the abnormal proliferation of fibroblast-like synovial cells (FLS), a common occurrence in RA. Under normal conditions, molecular mechanisms in human joints regulate FLS turnover, ensuring apoptotic clearance without excessive accumulation (1). Conversely, pathological conditions can disrupt these processes, leading to carcinoid-like deposits of FLS due to aberrant cell death dynamics. Notably, apoptosis and necroptosis serve as the principal cellular death pathways in this context (2). Apoptosis acts as a regulatory mechanism for necrotic cell death, potentially triggered in its absence, whereas necroptosis represents a form of regulated necrosis, similarly inducible without apoptotic pathways (3). Tumor necrosis factor (TNF), an inaugural cytokine capable of inducing cellular necrosis, exerts pro-inflammatory effects that enhance cell activation, proliferation, and survival (4). As progenitors of a broad family of ligands, their diverse functions have a significant impact on cellular fate. It has been determined that receptor-interacting serine/threonine kinase 1 (RIPK1) mediates inflammation and cell death, facilitating both apoptosis and necroptosis in TNF-stimulated cells (5, 6). Moreover, TNF and nuclear factor-kappa B (NF-kB) signaling pathway performs vital functions in various cancers and immune responses (7, 8). *RIPK1*-regulated inflammatory signaling, apoptosis, and programmed necrosis play crucial roles in RA-FLS. The interaction of RIPK1 with TNF receptor 1 (*TNFR1*) activates the NF-κB inflammatory signaling cascade, leading to RA inflammation. Modulation of RIPK1 activity affects FLS survival and death, thus influencing the intensity and duration of inflammation (9). *RIPK1* may further regulate the physiological and pathological functions of RA-FLS by interacting with other signaling molecules, such as MAPK. These interactions influence cell proliferation, migration, and secretion of inflammatory mediators, thereby playing a key role in the pathogenesis of RA (10). Although ubiquitination is an integral component of these signaling cascades, the ubiquitination-mediated proteasomal degradation pathway (UPP) has a vital part in the pathophysiology of RA by controlling the degradation of proteins associated with the inflammatory process. This pathway could affect the levels of inflammatory factors and other proteins, thereby influencing the inflammatory and immune responses in RA (11–14).

Ubiquitination, a vital post-translational modification, includes ubiquitin (Ub) and its target protein forming a covalent binding. Three different enzymes must function in succession for this process to occur, E1 (ubiquitin-activating enzyme), E2 (ubiquitin-conjugating enzyme), and E3 (ubiquitin ligase) (15, 16), culminating in the formation of an isopeptide bond between the C-terminal glycine (Gly76) of Ub and a lysine residue on the target protein (16). In rheumatoid arthritis (RA), ubiquitination influences immune cells and inflammatory responses by altering critical inflammatory signaling pathways, including NF-κB. For example, synoviolin 1 (*SYVN1*) is a novel pathogenic factor in RA, inherently an E3 ubiquitin ligase, highly expressed in the synovial tissues of RA patients and mice (17). *SYVN1* elevates quantities of inflammatory cytokines like TNF-α and IL-1β, inhibits apoptosis of synovial cells in collagen-induced arthritis in mice, and significantly promotes synovial tissue proliferation (18). Furthermore, midline 1 facilitates the progression of RA by regulating the proliferation, migration, invasion, and inflammatory activities of FLS through ubiquitin-mediated proteasomal degradation of DPP4 (19). While TNF-α inhibitors have markedly revolutionized the management of RA, not all patients exhibit responsiveness, and a significant majority experience relapse upon cessation of therapy (20). The clinical use of TNF inhibitors still needs to be further improved, in which case ubiquitination could potentially be used as an adjuvant therapeutic strategy. Ubiquitination can directly affect specific signaling proteins within the TNF receptor complex, promoting their degradation and thereby mitigating TNF-induced inflammatory responses. It can also specifically degrade excessively active inflammation-related proteins without completely suppressing the immune system, potentially reducing side effects of TNF inhibitors, such as immunosuppression and infection risk (21).

As E3 ubiquitin ligase, cellular inhibitor of apoptosis protein 1 (cIAP2), and cellular inhibitor of apoptosis protein 2 (cIAP2) are involved in the proliferation and survival of FLS by regulating TNF receptor signaling and inflammatory cytokine production to influence the immune response to RA, and interacting with apoptosis-related signaling pathways to prevent apoptosis (22). In addition, cIAP1 and cIAP2 play roles in the assembly of inflammasomes, regulating the release of inflammatory mediators and affecting the function of immune cells (23). Notably, cIAP2 is encoded by the *BIRC3* gene. Studies have found that *BIRC3* mediates NF-κB activation in various regions of RA synovium, promoting inflammation and disease progression. *BIRC3* also regulates inflammation and apoptosis in RA-FLS, indicating its dual role in promoting FLS survival and inflammatory responses (24). This review comprehensively analyzes the involvement of E3 ubiquitin ligases cIAP1/2 in RA-FLS, delving into the significance of cIAP1/2 in the regulation of TNF-induced cell death pathways and RA pathophysiology, emphasizing their roles as E3 ubiquitin ligases. Additionally, we highlight the potential clinical significance of the *BIRC3* gene, which encodes cIAP2 protein, as a therapeutic target, offering new perspectives for RA treatment.

## 2 Inhibitors of apoptosis proteins

CIAP1 and cIAP2 are members of the inhibitor of apoptosis protein (IAPs) family and play critical roles in regulating various cellular processes (25). IAPs, akin to their counterparts in baculovirus-infected mammalian cells, play pivotal roles in cellular regulation, including apoptosis, proliferation, and differentiation. This protein family includes X-linked IAP (*XIAP*), cellular IAPs 1 and 2 (cIAP1 and cIAP2), neuronal apoptosis inhibitory protein (*NAIP*), and baculoviral IAP repeat containing 5(*BIRC5*) (26, 27). IAPs are central to the modulation of several cellular processes, including signal transduction, cytokine production, and cell survival, thereby influencing both innate and adaptive immune responses. The E3 ubiquitin ligase activity of XIAP and cIAP1 primarily governs immune regulatory functions by targeting key signaling pathways such as NF-κB and mitogen-activated protein kinase (MAPK). Additionally, *NAIP*, cIAP1, and cIAP2 orchestrate inflammasome assembly, which is crucial for innate immune response (28). The hallmark of the IAP family is the baculovirus IAP repeat (BIR) domain, which is essential for protein-protein interactions (29, 30). In addition to the BIR domain, IAPs also possess other significant domains, including the C-terminal ubiquitin-conjugated (UBC) domain, caspase recruitment domain (CARD), and C-terminal RING zinc-finger domain, facilitating a range of functional interactions (29, 31). Originally characterized as caspase inhibitors, mammalian IAPs (cIAP1, cIAP2, and XIAP) also modulate apoptosis via E3 ubiquitin ligase activity (27). Specifically, cIAP1 and cIAP2 are integral to the receptor complexes of the TNF receptor family members, modulating signal transduction via ubiquitination of associated proteins. This includes their recruitment to TNFR1 through the TNF receptor-associated death domain (*TRADD*)/TNF receptor-associated factor 2 (*TRAF2*) pathway, leading to the ubiquitination of *RIPK1* within the TNFR1 complex (32, 33). The absence or inhibition of cIAP1 and cIAP2, particularly by the second mitochondrial activator of caspases (SMAC), renders cells vulnerable to TNF-induced apoptosis by disrupting NF-κB-mediated survival signals and facilitating the formation of pro-apoptotic TNFR1-induced complex II or *RIPK1*/*RIPK3*-dependent apoptosis (34, 35).

## 3 TNF-related pathway of cell death induced by cIAP1/2

### 3.1 TNFR1-induced complex

**Figure 1:**
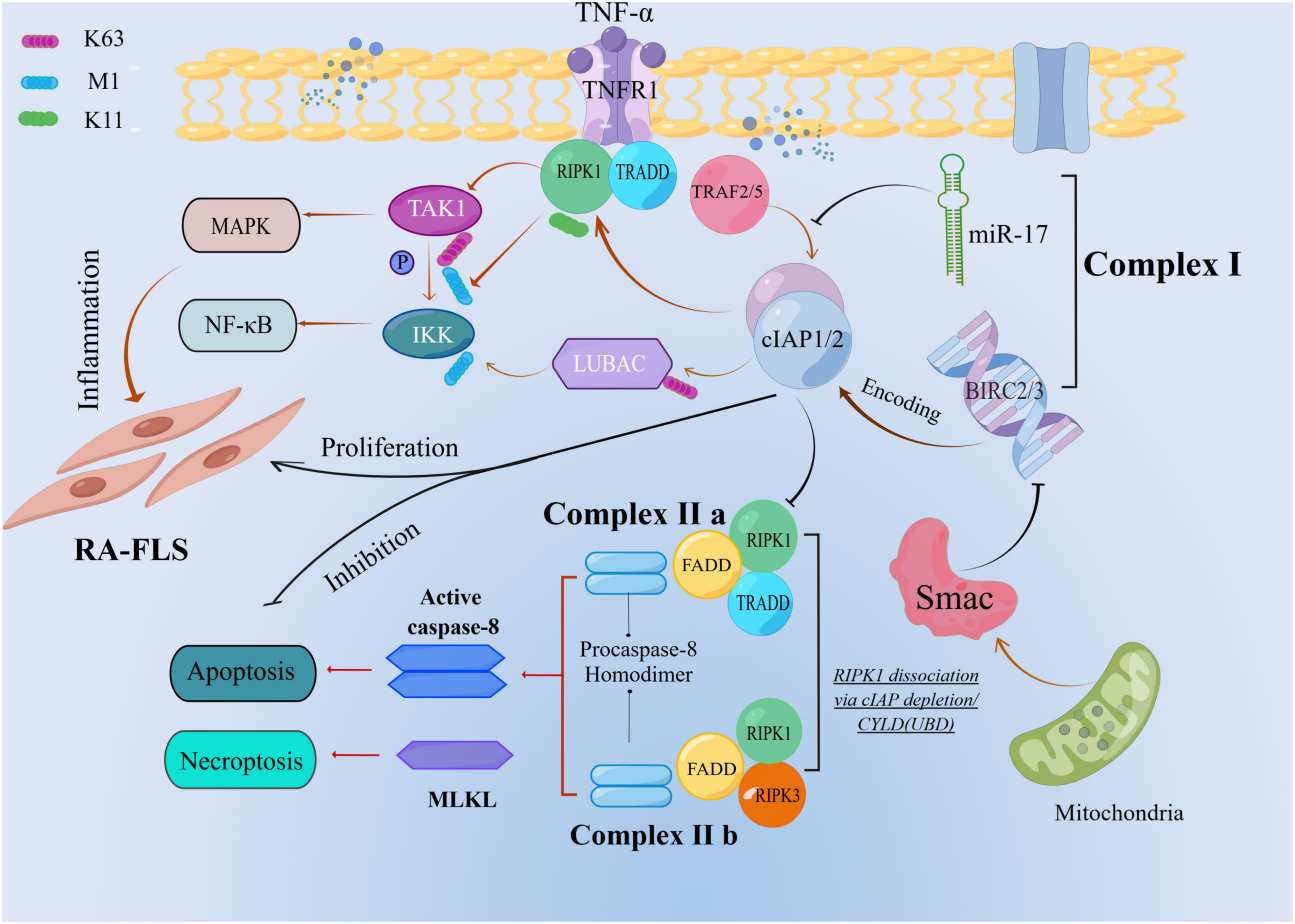
Ubiquitin-mediated regulation of TNFR1 apoptotic signaling. The cIAP1/2 mediates a series of ubiquitination in complex I. When *RIPK1* dissociation via cIAP depletion or *CYLD* is deubiquitinated, complexIIb is formed, which leads to apoptosis.

Binding of TNF to its receptor TNFR1 catalyzes the immediate assembly of the TNFR1 signal complex (TNFR1-SC), previously known as the TNF receptor 1 signaling complex (TNF-RSC). This complex incorporates *TNF*, TNFR1, *TRADD*, *RIPK1*, TNF receptor-associated factor 2 (TRAF2), cIAP1/2, linear ubiquitin chain assembly complex (LUBAC), inhibitor of kappa B kinase (IKK), and TGF-beta activated kinase 1 (TAK1) binding protein (TAB)-TAK complex (32). The integration of TAB-TAK and IKK complexes into TNFR1-SC relies on the recognition of lysine 63 (K63) and methionine 1 (M1) ubiquitin bonds by TAB2/3 and the NF-κB essential modulator (NEMO), respectively (36–38). The TAB-TAK complex requires only a K63 chain for recruitment, whereas the IKK complex requires both the K63 and M1 linkages. cIAP1/2, which serves as a crucial intermediary, facilitates this process by ubiquitinating several components of Complex I with K63-linked chains, including *RIPK1*. Subsequent recruitment of LUBAC enhances the M1-linked chain modification on *RIPK1 (* 39–41). These modifications recruit the TAK1 and IKK complexes, activating the MAPK and NF-κB pathways. Additionally, cIAP1 enhances IKK complex recruitment through the K11-linked chain modifications of *RIPK1 (* 42). Complex I represents the primary assembly in this signaling cascade. However, if *RIPK1* is deubiquitinated by CYLD lysine 63 deubiquitinase (*CYLD*) or remains unubiquitinated during Complex I formation (e.g., due to IAP depletion) (43), Complex IIa or IIb is generated, leading to apoptosis or necroptosis based on cellular conditions (44). Contrary to cIAP1/2’s supportive role in TNFR1 signaling, its function in other TNF receptor family members, such as the tumor necrosis factor-like weak apoptosis inducer (TWEAK) and CD40 ligand (CD40L), is inhibitory. cIAP1/2, in conjunction with *TRAF2* and *TRAF3*, suppresses alternative NF-κB pathways through ubiquitination of lysine 48 (K48) linkages and degradation of NF-κB-inducing kinase (NIK), in the absence of ligand stimulation (28) (**Figure 1**).

### 3.2 TNF-related apoptosis-inducing ligand receptor–induced complex

**Figure 2:**
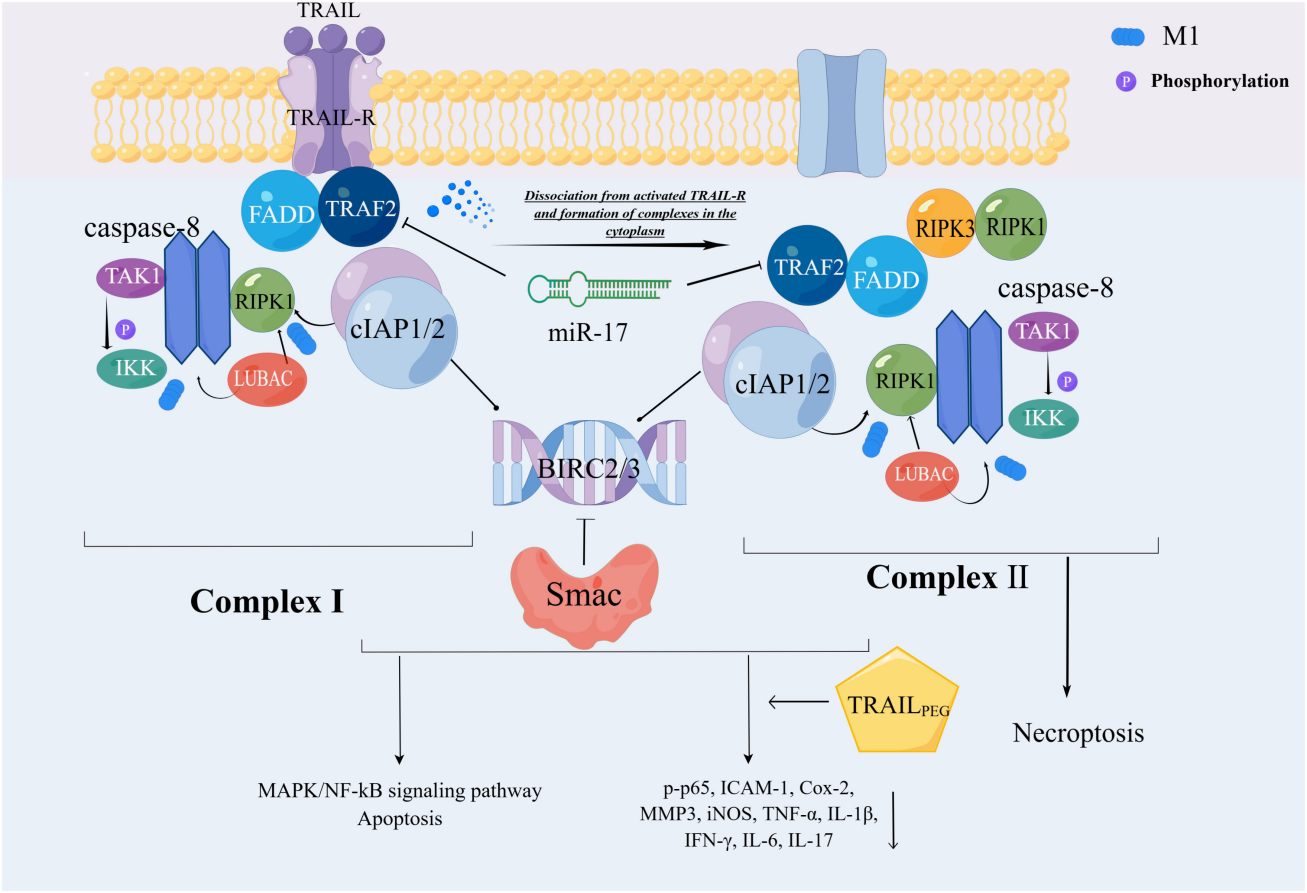
Ubiquitin-mediated regulation of TRAIL-R cell death signaling pathway. The composition of complexes I and II is relatively similar, with complexII derived from I detached from activated TRAIL-R. CIAP1/2 is involved in the ubiquitination of this process. Through the M1 chain, cIAP1/2 indirectly catalyzes the functional activation of caspase-8, which ultimately leads to the MAPK/NF-kB signaling pathway or apoptosis. This series of reactions is manifested in both complexes, but only complex 2 causes necroptosis.

TRAIL (tumour necrosis factor-related apoptosis-inducing ligand (TRAIL), also known as the APO-2 Ligand (*APO2L*), belongs to the family of tumor necrosis factors. The activation of TRAIL-R has been shown to induce the formation of complexes containing proteins similar to those induced by TNFR1, which are regulated by ubiquitin. In TRAIL-R signal transduction, pro-apoptotic proteins (FADD and Caspase-8) are first recruited to TRAIL-R and can be used as scaffolds for anti-apoptotic protein recruitment (*RIPK1*, *TRAF2*, cIAP1/2, LUBAC, TAK1, and IKK complexes) (45–47). The compound mentioned earlier is called complex I. The composition of complex **II** is very similar to that of **complex I**. It is generally believed that the former dissociates from activated TRAIL-R and forms cytoplasmic complexes (48). TRAIL-R-induced complexes **I** and **II** can activate NF-κB, MAPK pathway, and apoptosis, but only complex **II** can activate necroptosis (45). E3 ligase not only regulates apoptosis, but also the gene activation output of the TRAIL signal by participating in the two core signal components of TRAIL-induced gene activation, caspase-8 and *RIPK1 (* 47, 49). This process involved cIAP1/2. The E3 ligase cIAP1/2 is recruited to two TRAIL signaling complexes in a FADD-caspase-8-dependent manner. Furthermore, *TRAF2* and cIAP1/2 both promote TRAIL- and CD95L-mediated gene activation (47, 49–51). In the context of TNF signaling, TRAF2-cIAP1/2-mediated ubiquitination of RIPK1 promotes NF-kB activation (32, 42, 52, 53), the TRAF2’s gene activation function is dependent on its ability to recruit cIAP1/2 (33). Consistent with the activation of *TRAF2* as a TRAIL signal transduction scaffold, cIAP1/2 depletion strongly reduced *RIPK1* ubiquitin, IKK recruitment, NF-kB activation, and inactivation of TRAIL-mediated cytokine secretion, whereas *TRAF2* recruitment remained unaffected (49, 54). As previously demonstrated for TNF signal transduction, *TRAF2* may promote TRAIL-induced cytokine production by acting as a recruitment platform for cIAPs (33). Through an unknown mechanism, cIAP1/2 is also required downstream of *TRAF2* to recruit LUBAC to track complex I (49). LUBAC mediates TNFR1-SC stabilization via linear ubiquitination of *TRADD*, *RIPK1*, NEMO, and TNFR1 and is critical for TNFR1-induced gene activation signaling (40, 55). TRAF2 depletion strongly reduced *RIPK1* ubiquitination, IKK recruitment, NF-kB activation, and TRAIL-mediated cytokine secretion, whereas *TRAF2* recruitment was unaffected (49, 56) (**Figure 2**).

Although several recent studies have demonstrated that the mechanism by which TRAIL promotes RA inflammation does not depend exclusively on cell death, TRAIL regulation of RA inflammation is mainly due to its ability to promote apoptosis of synoviocytes and infiltrating lymphocytes (57). TRAIL produces various signals. TRAIL can induce migration, proliferation, and cytokine production in both cancerous and non-cancerous cells, in addition to inducing cell death via apoptosis and necroptosis. As a result, uncovering the mechanisms that regulate the complex balance between these various outputs can help us better understand TRAIL’s role of TRAIL in tissue homeostasis, immunity, and cancer. Previous animal experiments have confirmed that TRAIL_PEG_ ameliorates arthritis severity and significantly reduces the accumulation of inflammatory molecules (p-p65, ICAM-1, Cox-2, *MMP3* and iNOS), pro-inflammatory cytokines (TNF-α, IL-1β, IFN-γ, IL-6, IL-17) and activated macrophages (58), all of which have been shown to play a cellular and cellular component in important role in the pathogenesis of RA. The role of TRAIL in the mechanism of RA-FLS apoptosis has also been confirmed in several studies, and this role correlates with disease severity in a cell cycle-dependent manner (59–61). Blocking the expression of TRAIL and cIAP can significantly promote the apoptosis of FLS (59–61). In addition, ubiquitin and deubiquitination are key regulators of immune receptor signal transduction (45).

In comparison to TRAIL-R, the precise space and time positions of proteins in the TRAIL-R signal cascade are not well defined in TNFR1. However, it is unclear what determines whether complex I/II induces non-apoptotic or apoptotic signal transduction. However, ubiquitination of the M1 connection of Caspase-8 by LUBAC inhibits its activity (49), which may promote the spread of survival and inflammatory pathways that are usually inhibited by Caspase-8 activity (47, 62).

## 4 MicroRNA inhibits cIAP1/2 expression

In RA, both serum and synovial fluid (SF) samples, as well as synovial tissues and the serum and joints of rats with adjuvant arthritis, exhibit notably reduced expression of miR-17. Investigations revealed that miR-17 diminishes the expression of *TRAF2*, a cellular inhibitor of cIAP1 and cIAP2, in RA SFs when stimulated by TNF-α. Moreover, restoration of miR-17 activity enhanced the polyubiquitination of lysine 48 (K48) linkages of *TRAF2*, cIAP1, and cIAP2 in these cells, as determined by immunoprecipitation analyses. This modulation by miR-17 leads to the destabilization of TRAF2, impairing its ability to associate with cIAP2. Consequently, TNF-α suppresses the nuclear translocation of NF-κB p65, c-Jun, and signal transducer and activator of transcription 3 (*STAT3*) prompted by TNF-α. This molecular alteration results in decreased production of IL-6, interleukin-8 (IL-8), matrix metalloproteinase-1 (*MMP1*), and matrix metalloproteinase-13 (*MMP13*) in human RA SF, underscoring the pivotal regulatory role of miR-17 in inflammatory and degradative pathways in RA (63).

Besides RA-FLS, miRNAs also affect cIAP2 expression in other diseases. Studies have shown that *BIRC3* mRNA is upregulated in hepatocellular carcinoma (HCC) tissues, and the high expression of cIAP2 encoded by *BIRC3* promotes HCC proliferation and migration. Although miR-124 was underexpressed in HCC tissues and cell lines, *BIRC3* was identified as its target gene. Overexpression of miR-124 could target BIRC3, thereby decreasing cIAP2 protein levels and inhibiting HCC proliferation and migration through regulating the NF-κB signaling pathway (64). Circular RNA (circRNAs) are also involved in gene regulation. The oncogenic circular RNA hsa_circ_0070039 (circNUP54) is significantly upregulated in HCC. This interaction stabilizes downstream *BIRC3* mRNA by binding to the 3’-UTR region, thereby promoting HCC progression via the HuR/BIRC3/NF-κB axis (65). Furthermore studies on oral squamous cell carcinoma (OSCC) have shown that silencing circDOCK1 and upregulating miR-196a-5p results in increased apoptosis and decreased *BIRC3* formation (66).

## 5 Smac mimetics mediate cell death through degradation of cIAP1/2

IAPs, especially cellular cIAP1/2 and XIAP, can prevent cell death by preventing the activation of caspase-8 or inhibiting the activity of caspases-9,-3 and-7 (67–69), respectively. The E3 ubiquitin ligase domain of cIAP1/2 promotes cIAP1/2 and proteasome-dependent degradation (70, 71). A second mitochondria-derived caspase activator (Smac/DIABLO), which, along with cytochrome C, is released from the mitochondria under the induction of an inherent apoptotic pathway in response to stimuli such as genotoxic stress, relieves the inhibition of apoptosis by IAPs (72, 73). Direct binding and blocking of the interaction of XIAP with caspase-9,-3, and-7, as well as inducing proteasomal degradation of cIAP1 and cIAP2, are the main mechanisms by which Smac mimetics cause cell death (34, 35, 74, 75). The degradation of cIAP1/2 allows the release of *RIPK1* from TNFR1 and then incorporates the complex associated with caspase-8 and the fas-related death domain, which can promote cell death and cover the survival-promoting effect of NF-κB signaling (32, 76, 77). This means that any cell expressing TNFR1 should respond to Smac mimic/TNF-α therapy because the expression of TNFR1 is very common in all types of cancer cells (78). The E3 ubiquitin ligase activity of cIAP promotes cancer cell survival (32, 35, 74, 76). Furthermore, removing cIAP from cancer cells can alter cytokine signaling by converting pro-death signals into inflammatory signals, resulting in cell death via apoptosis or necroptosis (46, 79). *BIRC3* (cIAP2) is thought to be a member of the IAP family of proteins that regulate cell death and survival, according to Bai et al. (80). Previous studies have shown that *BIRC3* (cIAP2) promotes survival and anti-apoptosis in cancer cells and is a therapeutic target of the drug family known as “Smac mimic” (81), although not all cases support this view (81). Owing to its dual function in TNF, cIAP’s regulatory role of cIAP in cancer and the immune system is critical. Abnormal expression results in uncontrollable outcomes. In the field of cancer applications, the Smac simulator has been used in clinical trials of hematological and solid cancers, but unfortunately, the results so far are inconsistent and limited (82). The role of the Smac simulator in the treatment of RA is theoretically applicable (83). However, thus far, there has been little research on Smac simulations in RA-FLS. *In vitro*, it has been confirmed that *BIRC3* has an abnormally high expression in RA-FLS compared to OA. Simultaneously, given the carcinoid proliferation of FLS and the similar TNFR1 signal transduction mechanism of cIAPs in RA, Smac mimetics may be of breakthrough significance in the treatment of RA.

## 6 Discussion

Rheumatoid arthritis is a chronic inflammatory autoimmune disease characterized by the infiltration of immune cells into the synovium, leading to the release of inflammatory cytokines and subsequent tissue damage (84). In addition to inflammation, fibroblast-like synoviocytes, the principal effector cells in RA, also exhibit tumor-like proliferation and invasiveness. Therefore, controlling inflammation and inhibiting FLS proliferation are crucial elements in the treatment of RA (85). CIAP1 and cIAP2, E3 ubiquitin ligases, play critical roles in the regulation of *TNFR1* and TRAIL-R signal transduction, ensuring effective signal propagation. These proteins modulate various NF-κB pathways, which are pivotal for controlling diverse cellular inflammatory and immune responses. Although cIAP1 and cIAP2 are frequently discussed collectively because of their overlapping functions in ubiquitination across different signaling pathways, their specific roles in modulating cell death in distinct cell types and tissues remain poorly understood (86). In certain pathological conditions, cIAP1 has been shown to mediate disease progression through its regulatory effects on TNF signaling, whereas cIAP2 does not participate in this process (42, 87). Conversely, cIAP2 exhibits a unique therapeutic mechanism distinct from that of cIAP1 in the context of RA, highlighting the differential contribution of these proteins to disease pathology and treatment (88). In addition, within the array of death receptor (DR) ligands, TRAIL has garnered significant interest because of its structural homology with CD95L (89, 90) and its distinct ability to selectively eliminate cancer cells without notable systemic toxicity (91, 92). This characteristic has spurred the development of TRAIL receptor agonists (TRAs), although their clinical trials have been discontinued because of issues with their efficacy (93). Consequently, the targeted modulation of *BIRC3* gene expression and ubiquitination processes in TNFR1 or TRAIL signaling mediated by *BIRC3* (cIAP2) present promising strategies for enhancing treatments for related disorders, including autoimmune diseases.

The *BIRC3* gene encodes the multifunctional protein cIAP2, which plays an important role in the regulation of various cellular processes including caspases, apoptosis, inflammatory signaling, immunity, mitogen-activated protein kinase signaling, and cell proliferation (94). We speculate that increased expression of *BIRC3* in RA promotes FLS survival and contributes to the inflammatory response. Inhibition of *BIRC*3 expression can reduce the secretion of inflammatory cytokines by RA FLSs under both basal and inflammatory conditions, and impede their proliferation. Existing evidence has demonstrated that *BIRC3* plays a crucial role in promoting cancer cell survival and inhibiting apoptosis (95). The interaction between *BIRC3*, MAP3K14, and the NF-κB pathway highlights the impact of *BIRC3* inactivation on tumor cells dependent on this pathway (96). For example, in colorectal cancer cells, overexpression of *BIRC3* can lead to the activation of receptor-interacting serine/threonine protein kinase 2 (*RIPK2*), which promotes the ubiquitination of *IKBKG*, thereby inhibiting *IKBKG* protein expression and enhancing the expression of NF-κB subunits p50 and p65 (97). Recent studies have established a correlation between Fusobacterium nucleatum (Fn) and the occurrence and development of colorectal cancer (CRC), particularly noting that Fn infection upregulates *BIRC3* in CRC cells via the TLR4/NF-κB pathway (98). Moreover, the upregulation of *BIRC3* reduces the responsiveness of CRC cells to 5-fluorouracil (5-Fu), suggesting that targeting *BIRC3* may offer a promising strategy to alleviate chemotherapy resistance in advanced CRC (99).

Additionally, *BIRC2/BIRC3*, in complex with TNFR2 and TRAF2, activates the NF-κB and MAPK signaling pathways, which are crucial for inflammation and cell survival (24). Combining anti-tumor necrosis factor therapy with cIAP1/2 inhibitors by blocking the *BIRC2/BIRC3*-mediated apoptotic pathway has the potential to improve therapeutic efficacy and effectively reduce symptoms and disease progression in rheumatoid arthritis. According to researches, glioblastoma (GBM) is a highly malignant brain tumor characterized by elevated *BIRC3* expression, which is associated with tumor progression from low to high differentiation and resistance to temozolomide (TMZ) and radiation therapy, ultimately leading to reduced patient survival rates (100). Inhibiting the E3 ubiquitin ligase activity of *BIRC3* can increase tumor cell sensitivity to radiotherapy and chemotherapy, and developing *BIRC3* inhibitors may offer a promising approach for treating glioblastoma. Currently, small molecule inhibitors or antibodies targeting *BIRC3* are being developed as potential treatments to disrupt its E3 ubiquitin ligase activity and induce tumor cell apoptosis (101). A comprehensive study of the specific mechanisms of *BIRC3*, including its role in cell signaling, regulation of apoptosis, and evasion of immune responses, is crucial for identifying new therapeutic targets and strategies.

Although *BIRC3* inhibitors show promise in treating rheumatoid arthritis, potential side effects must be carefully considered, such as inducing apoptosis in normal cells dependent on *BIRC3*-regulated pathways and triggering adverse immune responses due to its role in immune regulation. FLS may activate other anti-apoptotic mechanisms, such as upregulating myeloid cell leukemia1(*MCL1*), thereby reducing sensitivity to *BIRC3* inhibitors (102). Furthermore, changes in the surrounding microenvironment, such as alterations in immunogenicity and extracellular matrix composition, may also affect the efficacy of these inhibitors.

This review discusses the critical roles of the E3 ubiquitin ligases cIAP1/2 in mediating cell death pathways induced by TNF and promoting abnormal proliferation of fibroblast-like synoviocytes in RA, emphasizing the importance of the *BIRC3*-mediated ubiquitination process and its potential as a therapeutic target. However, the review has several limitations. Primarily, it focuses on molecular and cellular mechanisms due to the scarcity of clinical studies on *BIRC3* in RA, lacking support from large-scale, multicentric clinical trials. Additionally, it does not cover longitudinal studies to track the long-term effects of *BIRC3* inhibition on RA, which could provide deeper insights into the durability of treatment effects and potential development of resistance or adverse reactions. Although the article mentions several signaling pathways affected by *BIRC3*, it does not sufficiently explore alternative pathways that might compensate for *BIRC3* inhibition, which could aid in understanding potential resistance mechanisms and refining therapeutic strategies. Due to the limited research on targeting *BIRC3*, there is a lack of discussion on the potential side effects and safety concerns of *BIRC3*-targeted therapies. Finally, a more robust comparative analysis is needed, especially comparing the role of BIRC3 in RA with other autoimmune diseases, to provide a broader perspective on its specific functions and potential as a therapeutic target.

## Glossary

RA: rheumatoid arthritis
BIRC3: baculoviral IAP Repeat Containing 3
TNF: tumor necrosis factor
CIAP1: cellular inhibitor of apoptosis protein 1
CIAP2: cellular inhibitor of apoptosis protein 2
FLS: fibroblast-like synoviocytes
RIPK1: receptor interacting serine/threonine kinase 1
Ub: ubiquitin
Gly76: C-terminal glycine
TNF-α: tumor necrosis factor alpha
IAPs: Inhibitors of apoptosis proteins
FADD: X-linked IAP
NAIP: neuronal apoptosis inhibitory protein
BIRC5: baculoviral IAP repeat containing 5
NF-kB: nuclear factor-kappa B
MAPK: mitogen-activated protein kinase
BIR: baculovirus IAP repeat
UBC: C-terminal ubiquitin-conjugated
CARD: caspase recruitment domain
TRADD: TNF receptor-associated death domain
SMAC: second mitochondrial activator of caspases
TNFR1-SC: TNFR1 signal complex
TNF-RSC: TNF receptor 1 signaling complex
TRAF2: TNF receptor-associated factor 2
LUBAC: linear ubiquitin chain assembly complex
IKK: inhibitor of kappa B kinase
TAK1: TGF-beta activated kinase 1
K63: lysine 63
NEMO: NF-κB essential modulator
CYLD: CYLD lysine 63 deubiquitinase
CD40L: CD40 ligand
TWEAK: tumor necrosis factor-like weak apoptosis inducer
K48: lysine 48
RING: really interesting new gene
NIK: NF-kB-induced kinase
TRAIL-R: TNF-related apoptosis-inducing ligand receptor
APO2L: APO-2 Ligand
TRAIL: tumour necrosis factor-related apoptosis-inducing ligand
ICAM-1: intercellular cell adhesion molecule-1
MMP3: matrix metallopeptidase 3
iNOS: inducible nitric oxide synthase
IL-1β: interleukin 1 beta
IFN-γ: interferon gamma
IL-6: interleukin 6
IL-8: interleukin-8
IL-17: interleukin 17
STAT3: signal transducer and activator of transcription 3
MMP-1: matrix metalloproteinase-1
MMP-13: matrix metalloproteinase-13
DR: death receptor TRAs, TRAIL receptor agonists
MCL1: myeloid cell leukemia1
GBM: glioblastoma
